# Principles of Medicine: Comprising General Pathology and Therapeutics, and a Brief General View of Ætiology, Nosology, Semeiology, Diagnosis, and Prognosis

**Published:** 1844-04-01

**Authors:** 


### Medico-Chirurgical Review,

### No. LXXX.

**[No. 40 of a Decennial Series.]**

JANUARY 1, TO APRIL 1, 1844.

### PRINCIPLES OF MEDICINE: COMPRISING GENERAL PATHOLOGY AND THERAPEUTICS, AND A BRIEF GENERAL VIEW OF ÆTIOLOGY, NOSOLOGY, SEMEIOLOGY, DIAGNOSIS, AND PROGNOSIS. By *Charles J. B. Williams,* M.D. London, 1843.

IT is not a little extraordinary, that whilst the most valuable improvements are being daily made in the preparatory sciences of Anatomy, Physiology, Chemistry, &c., no work has appeared until within the last few years, calculated to point out to the student the bearings of these sciences on practical medicine, and to render the discoveries which have been made in the former applicable to the advancement and improvement of the latter. In fact, we possessed until lately no work which treated fully of those general principles in the nature and treatment of disease, which are really fundamental to practical medicine. And yet what are the objects which the student has in studying anatomy and physiology? is it not for the purpose of making his knowledge of these preliminary sciences fundamental to that of medicine, so that starting from the knowledge of the healthy body, as derived from them, the transition might be easy and intelligible to the study of disease.

By general principles in medicine are to be understood those general truths and doctrines, which have been ascertained and established by the continued observation of attentive minds throughout the entire progress of medicine as a science. The sources whence the principles of medicine should be derived are, 1st, a knowledge of animal structure and function (anatomy and physiology), and 2d, a knowledge of those agents which cause and remove disease. Under the head of principles of medicine are comprised those leading and general facts and doctrines regarding disease and its treatment, which are applicable not merely to individual cases, but to groups or classes of disease. Thus, for instance, there are facts and doctrines, common to all inflammations, to all hæmorrhages, and to all dropsies. By combining these generalities into one comprehensive principle, we help the memory, and avoid needless repetitions. This branch of medicine is called General Pathology. An acquaintance with these principles will enable the student to see with intelligence—to read, understand, and interpret the book of Nature, when it is laid before him; it will, in fact, accomplish that which is the ultimate end and aim of all our preparatory studies in medicine; it will qualify him for practising his art with credit to himself, and benefit to his patient.

It may appear strange, and yet it is not the less true, that different writers on the subject of General Pathology differ widely from each other as to that which should constitute this branch of medical science. Some have treated the subject as if it were identical with semeiology, or that department which treats of the phenomena of disease; some have made it to consist almost entirely of scraps of pathological anatomy, whilst others again, from their horror of systematizing and of introducing hypotheses and groundless conjectures into medicine, have altogether banished from their treatises on the subject any thing at all bordering on the process of induction, and have made what they call general pathology to consist of stale truisms and unconnected aphorisms. There are some who will have it that General Pathology should serve as a complement to Special Pathology, precisely as General Anatomy does to Special or Descriptive Anatomy. If by this we are to understand that the study of General Pathology is to follow that of Special Pathology, we must express our most unqualified dissent. In commencing our study of disease with that of General Pathology, we commence with that which is the more elementary, and consequently the more simple, and, having thus acquired a knowledge of the elements of disease, we are prepared to encounter the study of it in its more complex form. The many advantages of proceeding in this way over the method of studying special pathology and nosology, as is the usual practice, will appear evident, by our contrasting a student educated in general principles with the nosological student, when they are severally brought to the bed-side of a patient. The latter comes to the patient, crammed with the nosological definitions of diseases current in the schools. In some well-marked and fully developed acute cases, where he cannot mistake, he may go on very well; but, in the more ordinary description of cases, in their early stages and endless variations, the phenomena do not correspond with any of his definitions; they frequently change their character in a manner altogether unaccountable to him; he is puzzled, his diagnosis fails him, his prognosis is proved wrong; thus disappointed in the failure of his nosological learning, he mistrusts it every day more and more, and at length falls into a routine of empirical practice. Let us now consider the pathological student. He has learnt to trace symptoms to their causes. He meets, we will suppose, a patient, who complains of violent headache and giddiness; having been taught by anatomy the peculiarities of the circulation in the head—and by physiology confirmed by clinical observation, that the circulation may be similarly impeded by opposite causes, inanition as well as fullness—he is prepared to find out, through other symptoms, which is the cause of the headache in the case before him—and he adapts his remedies accordingly. Again, we will suppose a patient to present himself labouring under ascites; the nosological practitioner, who has been accustomed to treat symptoms, looks no farther than the ascites, and sets about treating his patient by purgatives and diuretics, and too often ineffectually; whilst the man well acquainted with the principles of General Pathology traces the collections of water (symptom) to its cause, (some organic lesion.) His pathology has taught him that ascites may depend on a variety of organic lesions; that it is sometimes referrible for its cause to disease of the heart or lungs, to disease of the liver or of the peritoneum itself, to disease of the kidneys, or to a depraved state of the blood. He then sets himself to find out by other symptoms which of these organs is at fault, and having ascertained it, he applies his remedies according to the nature of the case. Hence it is evident that the student should be constantly reminded that the practice of medicine is the useful application of all his previously-acquired knowledge. And yet how are we to account for the extreme reluctance and irksomeness, which students in general feel in prosecuting the study of practical medicine? The author thinks, and we fully concur with him, that it is to be attributed in a great measure to the manner in which this branch is generally taught. Instead of making the previously-acquired knowledge of anatomy and physiology fundamental to that of medicine, the usual course adopted by Lecturers is to plunge at once into the mazy thickets of inflammation and fever—subjects so complicated, so different from any thing taught by previous study, that anatomy and physiology afford but little help. This plan of proceeding is aptly compared by the author to a person beginning the study of mechanics with the steam-engine; or to the student of chemistry commencing with organic matter. The general result is, that where any distinct notion of disease is acquired by this injudicious course of proceeding, it is one not at all founded on previous physiological knowledge, but is a new idea altogether of disease, as an absolute, separate, independent thing—not a mere condition of altered function or structure, but some real being, whose character and history are to be detailed like that of a plant or an animal. The teacher of practical medicine should always keep in view the observation made by Herm. Boerhaave, in his *Inst. med. Docenti procedendum est a generalibus ad Singularia quæque.*

Without the connecting link of general pathology, practical medicine derives little or no aid from anatomy or physiology. Instead of being founded on them, it is studied and practised quite independent of a full knowledge of them, and is generally acquired in proportion as they are forgotten. This kind of practical medicine is much the same as that of old women and nurses; it consists chiefly of treating symptoms, or groups of symptoms, as may have been found useful in similar cases, without the trouble of enquiring after the causes of the symptoms or the seat of the disease. One of the great advantages of an acquaintance with general pathology is, that it furnishes the student *in limine*, at the very outset of his clinical career, with that sort of knowledge, which the mere symptom-treating practitioner has acquired, and that very imperfectly, after years of observation and experience. Sound principles of medicine are, in fact, the embodiment of the result of experience in disease, with a knowledge of structure and function in health. It is well observed by our author, that one of the greatest proofs of the practical utility of general pathology is the aid which it gives in the practice of clinical medicine. In fact, the leading rules of practice, those which guide the most experienced men, (although many are not aware of it,) are founded on general views of diseased function and structure, that is, General Pathology. Practical men, no matter how imperfect their education may have been, do not treat a disease by its name. They really act more on general ideas of disease than on their knowledge of any particular disease. Hence it will appear how important it is that those general views which are so practical, and so extensive in their application, should be well-founded and carefully studied, and that the leading doctrines of disease, should not be left to be picked up irregularly, from casual retrospects of study or experience, when they may be learned during the preparatory course of Clinical Education, as the very ground-work of practical knowledge. We cannot conclude these remarks, prefatory to our analysis of Dr. Williams' work, more appropriately than by quoting the following passage from Dr. M. Hall, *On the Mutual Relations between Anatomy, Physiology, Pathology, and Therapeutics.* "Every day Physiology becomes a more certain science, and more a science of phenomena and principles. Theory is taking the place of hypothesis, experiment and observation of conjecture. Every day, too, the bond which unites the science of physiology with the practice of medicine is drawn tighter. The public as well as the profession must be enlightened on these subjects. We shall then see empiricism disappear, while ignorance and mystery in medicine are dispelled together. There are no quacks among the engineers, because every one knows that an engine must be understood by him who would repair it. When this truth obtains with regard to medicine, then and not till then, will that most complicated of machines, the human frame, cease to be confided, in the derangements of its functions, or the diseases of its structure, to any one who is ignorant of the many springs of its action and principles of its composition."

We now proceed to our more immediate task—that of presenting an analytical view of the subjects treated in this work. The first Chapter treats of *Ætiology,* or the causes of diseases. This part of the work we shall pass over, there being nothing in it very particular, and shall proceed to an analysis of the Second Chapter, which commences with Pathology proper, or *Pathogeny.*

CHAP. II.—This chapter commences with the consideration of Pathology proper, scil. the nature and constitution of diseases. Disease is defined to be a change from the natural condition of the function and structure of the body; now, as the functions or structure are compound, it is obvious that we cannot obtain an accurate knowledge of the disease, until we have obtained that of the several elementary functions or structures affected. The pathologist should follow the example set him by the anatomist, physiologist, and chemist; he should study the constituent parts or *elements,* in disease, before he can understand their combinations—and yet how differently do men usually proceed in the study of pathology! they usually commence with the complex subjects of inflammation and of fever, before they have made themselves acquainted with the elementary properties of textures, or even of vessels. We have the healthy and diseased *primary or ultimate elements of structure*—muscular fibre, nervous matter, vascular fibre, and the elementary tissues of membranes, glands, &c.; as also the *primary elements,* healthy and diseased, of *function*

of these same structures,—irritability, tonicity, nervous properties, the power of secretion and nutrition; and, lastly, the constituents of the blood; we have also the *secondary* or *proximate elements* of disease; the different states of the blood-vessels, scil. anæmia, plethora, congestion, determination of blood, and inflammation; the different functions of the nervous system, sensation, volition, reflected excitement, sympathy and irritation; the secreting organs and membranes, with their relations to the vessels, the nerves, &c.; and, lastly, the elements of structural diseases. Such are the especial subjects of general pathology. Having now divided the elements of disease into primary or ultimate, and proximate, our author proceeds to the distinct and separate consideration of each, commencing with the primary elements of functional or dynamic diseases. Among the primary elements he first notices irritability, or the property of contracting on the application of a stimulus. This, Dr. Williams coincides with Haller, in considering a property of muscular fibre. The property may become excessive, constituting spasm or convulsion. Such excess may manifest itself in three ways:—1, by excessive strength; 2, by inordinate quickness; and, 3, by the unusual duration of the contractions. Excessive strength is exemplified in the violent action of the heart in excitement; or in the great muscular power of a delirious patient. It may arise from excessive stimulus;—or from muscles being over-fed with blood. Inordinate quickness of contraction constitutes mobility of muscle, and frequently co-exists with want of power in the contractions—it is exemplified in the irritable heart; in the quick nervous movements of irritable persons—in irritable bowels, and irritable bladder. The pathological cause appears to be either an undue flow of blood to the muscles, or an irregular distribution of nervous influence. The most remarkable instances are given in *convulsions* or *clonic spasms*—as in chorea, epilepsy, and convulsive hysteria. An inordinate duration of muscular contraction constitutes *tonic spasm*, or *cramp*, in which the contraction does not alternate with relaxation. The extreme example of this is tetanus. The chief remedies for these affections are blood-letting, revellents, narcotics, and antispasmodics.

Having thus considered muscular contractility in excess, the author next considers it in its state of deficiency. This state may be occasioned by over-exertion—want of due supply of blood—sedative poisons. These agents, when carried to an extreme degree, cause paralysis, or complete loss of irritability. Muscular irritability may be deficient in readiness to contract—this is exemplified by the sluggish movements of persons who have taken opium, and in the slow pulse caused by digitalis. Though muscular irritability may probably not be *derived* from the nervous system, it is evidently very much under its influence. The nerves are the proper medium by which the voluntary muscles act, as well as the involuntary. The remedies for defective irritability must vary with its cause; stimulants—electricity, &c. are the principal. We next come to Tonicity, or tone, by which is meant a tendency to slow, moderate contraction, not necessarily terminating in relaxation; a property which keeps the parts in a certain degree of tension. To a certain extent tonicity is affected by the same agents which excite irritability; temperature, however, seems to affect them differently. Tonicity may be excessive or deficient. Where it is excessive, the muscles are very firm—pulse strong, tense, and often slow—capillary circulation active—from the tense state of the vessels and skin, the several secretions are much diminished—this state leads to local congestion, active hæmorrhage or inflammation, apoplexy or gout. The remedies are relaxants—warm bathing—exercise—sudorifics—aperients, &c.

Defective tonicity is characterised by flabby muscles, incapable of much exertion. Heart is irritable—pulse soft and unsteady—it is frequently retarded. Want of tone in the stomach and intestines causes indigestion and constipation—the secretions also are scanty, depraved, or profuse and watery. A person, thus affected, has little power to resist the influence of depressing agents. The remedies for such a state are obviously those of a tonic character; among which may be mentioned the judicious application of cold.

Our attention is now directed to the properties of the nerves—and first, to the modifications of *Sensibility*—these modifications may depend either on disease of the centre, producing disorder of general sensibility, or on disease of one or more nerves, causing disorder of local sensibility. The diseases of general sensibility may consist in *excess*—*defect*—or *perversion*. Excessive sensibility is more or less present in the early stages of inflammation of the nervous centres. This excessive sensibility, however, is congenital in some persons. Such over-sensibility is generally conjoined with excess of irritability and want of tone. Other nervous functions, also, as sympathy and reflex action, are also often augmented or disordered. The pathological cause of increased general sensibility is probably an undue supply of blood to the posterior columns of the spinal marrow, and the parts of the cerebral mass concerned in sensation. We entirely coincide with Dr. Williams in his opinion, that the luxurious habits of the upper classes, with more excitement for the mind than for the body, and for the feelings than for the understanding, are well adapted to foster morbid sensibility. The remedial measures suited to the removal of this element of disease, are narcotics and anodynes—where it depends on vascular excitement of the nervous centres, the antiphlogistic treatment is most beneficial. Where excessive sensibility is accompanied with general debility, weak and slow pulse, and absence of heat of skin, country air, &c. in fact, general and strict attention to dietetics, are most to be recommended.

*Defective* general sensibility, in its extreme degree, is exemplified in coma from impeded circulation in the nervous centre, arising from pressure, or in consequence of narcotism. It may also arise from the blood becoming impure from the retention of excrementitious matters, as in suppression of urine. Persons so affected have but little irritability, and are remarkable for being exempt from many diseases, whilst they are very liable to others, such as depend on a plethoric state of the system. The remedies applicable in such a state depend on the cause—when it arises from plethora, depletion and derivation are indicated. After considering diseases of voluntary power, general and partial, our author proceeds to Diseases of Reflected and Sympathetic Nervous Influence.

According to the discoveries of Dr. Marshall Hall, the contractions of all the sphincters, of the œsophagus, glottis, &c. the regular action of the respiratory muscles seem to be sustained, independently of the will, by a nervous influence, conveyed by afferent nerves from the respective parts to the spinal marrow, and reflected from it through the efferent nerves to the muscles connected with these parts. Alterations in this nervous property oftentimes become elements of several diseases. We have instances of the *increase of this involuntary excito-motory power* in spasm of the throat, and sometimes of the sphincters, in certain nervous diseases, as tetanus and some hysterical affections. The hurried respiration, convulsive cough, violent retching, and hiccup, presented in many nervous diseases, may be, in part at least, traced to an undue influence of the excito-motory nerves of organic life. A similar exaltation of this function is exemplified in the voluntary muscles, when they are deprived of sensation and voluntary motion by disease in the brain. Thus, in paraplegia from disease in the upper part of the spine, the excito-motory power of the nerves of the lower extremities is exalted, and tickling, or mere touching the soles of the feet or legs, will produce convulsive motions, though all voluntary power or sensation be lost. Under the same head we may consider convulsions, which must be referred to an irritation of the true spinal system. This irritation may be *centric*, as in epileptic and apoplectic convulsions from disease in the head, and those from loss of blood; in which cases, the spinal and prolonged medulla being excited, the excito-motory influence radiates to the limbs and muscles generally; or it may be *excentric*, commencing at the extremities of some afferent nerve, which transfers it to the spinal centre, whence it is again reflected. Such are the convulsions arising from teething, uterine, intestinal, or renal irritation. *Partial spasms* caused by *reflected irritation* are exemplified in cramp in the legs from acrid matter in the colon, in diarrhœa and cholera; retraction of the testicle from calculus or inflammation of the kidney, &c. also in sneezing from irritation of the nerves, coughing from irritation of the glottis, retching from irritation of the fauces. Striking instances of reflected irritation are displayed in the involuntary muscles, the heart, and muscular fibres of the air-tubes and intestinal canal. Thus irritation in the stomach, intestines, or other viscera will cause irregular action of the heart. Spasm of the intestines in colic is brought on by reflex irritation from acrid matter in them. Intestinal irritation may also induce spasm of the bronchi. With respect to the causes of this excitement, it is sometimes referrible to an increased flow of blood through the spinal cord or its nerves, sometimes to mechanical irritation of the cord or its nerves, from the effect of tumors and spicula of bone in the cord, head, or in the course of the nerves—traumatic tetanus exhibits this nervous irritation in a frightful manner—accumulation by rest, also, will cause an increase of this property—the same effect may be produced by sedentary habits. *Defect* of the reflex power is exemplified in paralysis affecting the sphincters, eyelids, muscles of respiration, &c. When this becomes general, the result is fatal, because the respiration, deglutition, and other actions essential to life suffer. Hence it is, that apoplectic coma and narcotism prove fatal. Involuntary voiding the urine and fæces, and the breathing becoming irregular and gasping are indicative of a failure of the reflex power. With respect to remedial measures, when excessive reflex action depends on inflammation or congestion of the cord, the means to be employed are obvious. When the irritation is purely nervous, as in tetanus, or hydrophobia, sedatives are indicated, such as hydrocyanic acid, Indian hemp resin, &c. Stimulant antispasmodics, more especially in weak subjects, and in the absence of inflammation, are found to act as sedatives on the spinal-nerves. Not only motions, but sensations also may be reflected. Thus, touching the external auditory meatus causes a tickling sensation in the glottis. Congestion of the liver is sometimes accompanied by pain in the right shoulder-blade. In such cases the sensations are to be referred to an influence reflected probably from the spinal centre. Severe frontal headache may be occasioned by acrid ingesta.

We next come to the subject of the *Diseases of Secretion.* Variations in the process of secretion are oftentimes referrible to changes in the supply of blood sent to the secreting organ. The quantity or quality of the secretion will also depend on the quantity or quality of the blood. Affections of the nervous system, as also of the mind, may affect the process of secretion; mental agitation will produce diarrhœa—nervous excitement will occasion a large flow of limpid urine. The secretions may be excessive, defective, or perverted. Excessive secretion may have the effect of weakening the system by the drain it causes from the mass of blood—each secretion also may have peculiar effects, connected with its office and composition; these effects may be *forwards*, on the parts to which the secretion goes, and *backwards*, on the organ and the blood from which it is formed. The *forward* effects of an excessive secretion of bile depend on its stimulating properties. By irritating the intestinal tube it causes diarrhœa—an excessive secretion of mucus in the bronchi may occasion dyspnœa and cough. Excessive secretions of secreting organs may amount to a flux; whilst those from enclosed serous surfaces or cellular tissue constitute the various forms of dropsy. The *backward* effects of excessive secretion may be referrible to the *organ* and also to the *blood*—the effect on the organ will be that it may become torpid. Excessive secretion, if abounding in animal matter, may reduce the mass, and change the composition of the blood. The excessive secretion of bile or urine modifies the blood. In the case of the latter, a predominance of hydrogen and carbon would be left in the blood, whilst the excessive secretion of bile would leave a predominance of azote. The *remedies* of excessive secretion will depend on the cause—if it arise from determination of blood, depletion, derivation and evacuation must be employed—in such cases the excessive secretion should not be too hastily checked, as it may be a natural means of relief. Where the excessive secretion arises from nervous or other sources of irritation, it must be checked by means which act as general tonics or astringents, and by such also as act only on particular organs. Great advantage will also be derived from means which increase other secretions, by which means the balance may be restored. *Defective Secretion* of any natural or habitual discharge may cause plethora, which will be general, if the secretion be copious, and local, if it be inconsiderable. The morbid effects of defective secretion may be *forwards*, and *backwards*, as in the case of excessive secretion. With respect to the *backward* effects, for instance, of sudden suppression of urine or bile typhoid symptoms are observed to follow it, as also extreme depression and coma, which soon end in death. The excretions are defective in many idiopathic and symptomatic fevers, and several of the constitutional effects of these fevers are due to this circumstance. The remedies for defective secretion are to be regulated by the cause—where inflammation or congestion exists, depletion and derivation are indicated—where there is a defective supply of blood stimulants may help to restore the secretions. When the first disorder is in the secreting structure itself, the defect may be removed by agents which specifically augment the secretion. Thus mercury will increase the secretion of the liver; the various diuretics that of the kidneys, purgatives that of the intestines.

Perverted secretion often accompanies excess and defect of this process. Thus, in fevers, the secretions are altered as well as diminished. The quality of the urine often becomes much changed—full living and stimulating beverages will render it strong and acid; whilst low diet, great fatigue of body or mind, and chronic inflammation of the kidney, generally make it pale and alkaline. Generally speaking the remedies for perverted secretions are likewise those which increase the secretion. Where the perversion depends on altered circulation in the part, the use of tonics is indicated. Our author having now considered the vital properties of the elementary solids in their pathological relations now considers the elementary changes of the blood in disease. The blood consists of red particles, colourless globules, and liquor sanguinis, or, the latter being itself compound, the following may be set down as its composition. 1. Red particles. 2. Fibrin and colourless globules. 3. Albumen and other animal matter. 4. Oil. 5. Salts, and 6. Water. These elements may be present in excess, defect, and in a state of change.

*Red Particles.*—The red blood-discs are considered to be the part of the blood on which its vivifying properties chiefly depend. Their excess may therefore be supposed to cause general excitement of the vital properties of the system—they exist in large proportion in persons of a sanguine temperament—and more in males than in females. Andral and Gavarret detected an excess in the early stage of inflammations and fevers. The red particles are *defective* in persons of the lymphatic temperament; after great losses of blood—in chlorosis and other anæmic states—in scrofulous and tubercular diseases—in the latter periods of fever—in granular degeneration of the kidneys attended with dropsy. The signs of the defect are, as might be expected, paleness of parts naturally coloured with blood and sallow hue of the skin. The red particles are changed in some diseases, the colouring matter being much darker than usual, as in the worst forms of scurvy—in some malignant fevers it has been described as being pitchy black. In congestive typhoid fevers there appears to be an unnatural solution of the red particles. The black matter of melanosis seems to be the colouring part of the blood in an altered state. When the red particles are in *excess*, the most effectual *remedy* is blood-letting—low diet also will assist;—where the red particles are *deficient*, animal diet, exposure to air and light, with tonics generally; and more especially the preparations of iron.

*Fibrin.*—The fibrin is the part which causes the coagulation of the blood, and constitutes the buffy coat and coagulable lymph. It is deficient in new-born animals, but abundant in children, and in well-fed persons. An *excess* of fibrin and of the colourless or lymph globules, exists in in ammatory diseases, especially those of a sthenic character, and in acute reflumatism. *Defect of fibrin* is indicated by imperfect coagulation of the blood when drawn. Venous blood contains less fibrin than arterial. Great bodily fatigue and want of sleep expend the fibrin. In some instances the blood is found fluid in cases of death from poisoning and other sudden causes. In adynamic fevers, also, the blood is fluid and imperfectly coagulable. A defect of fibrin causes a tendency to hæmorrhages, generally of an asthenic kind. In such cases, wounds do not readily heal, nor fractures unite. The author observes that a certain spissitude in the blood is favorable to its transit through the hydraulic apparatus of the circulation; and that, when this is deficient, various irregularities in the distribution of the blood may occur.

Our author now passes in review the important morbid appearances presented by the buffy coat and contraction of the clot of blood. For valuable information on these several points we must refer to the original. We now pass on to the *Changes in the Blood by Respiration.* The conversion of venous into arterial blood comprises the absorption of oxygen, the removal of some carbonic acid, a slight increase of fibrin, &c. Each part of this process is probably concerned in fitting arterial blood for its function; the absorbed oxygen, by its affinity for the hydrogen and carbon of the blood and textures, aiding in those processes by which these are renovated in function as well as in structure and heat is evolved; the renewal of fibrin supplying the expenditure of the plasma; and the removal of the carbonic acid being the excretion of a noxious matter.

*Defect* of the change of the blood by respiration is an important element in disease, and constitutes a prominent feature of affections of the respiratory apparatus. This constitutes the essence of *asphyxia* or *apnœa.* The mischief arising from defective respiration varies according to the sudden or gradual supervention of the defect. Persons affected with extensive emphysema of the lungs are habituated to an imperfect state of respiration, as evidenced by a constant lividity of the lips and cheeks—such an appearance would be a sign of death in other persons. The chief cause of this difference lies in the fact, that the importance of the respiratory function varies under different circumstances. When the muscular parts of the body are in full activity, more breath is needed to remove from the blood the noxious effete matter, which always results from great exercise. In such a state the respiratory process cannot be abridged without serious disorder. The phenomena of asphyxia are compounded of—1, accumulation of blood in the venous system; 2, diminution of blood in the arterial system; and 3, deficiency of oxygen and excess of carbonic acid in the blood. These several conditions injure the vital functions, both by the want of a due supply of blood, and by the bad quality of that blood, which is injurious,—negatively for want of oxygen, and positively from its excess of carbonic acid and other excrementitious matters which are sedative. The symptoms induced are also of two classes—1, those implying failure of function, such as muscular debility, feeble action of the heart, coldness of the surface and extremities, and abolition of the senses and mental faculties; 2, those arising from congestion and the noxious influence of the black blood, such as palpitation, flashes in the eyes, noises in the ears, delirium, muscular spasms, &c. There is another mode in which the changes by respiration may become defective, that occurring gradually, or when the functions are not active. This may be seen when the defect is congenital, as in malformation of the heart, causing cyanosis, in which case some venous blood passes into the arteries—it is also seen where the defect is very gradually induced, as in emphysema of the lungs. The chief indication here, is to restore the respiratory function, where it is defective. The injurious effect of defective respiration may be diminished by lowering the activity of the functions—by enjoining complete rest of both body and mind—by warmth to the surface and extremities, whilst air is supplied cool and fresh to the face and air-passages—by sedatives which reduce the circulation and other functions to a lower standard. In extreme cases, stimulants may be required for the enfeebled circulation, and depletion to remove the engorgement of the venous system.

We shall now pass on to the *secondary or proximate elements of disease.* The class of *proximate* elements which have been most generally studied as the subjects of general pathology, are those affecting the circulation of the blood. The morbid conditions connected with *defect* and *excess of blood* in the vessels, come now to be considered under the divisions of *general* and *partial,* and as attended with an increase or diminution of the irritability and tone of the moving fibre. *Anæmia,* or, as it is sometimes called, *oligæmia,* is the name given to that condition of the system characterised by *deficiency of the blood.* It is often symptomatic of other diseases, but sometimes occurs without any other known disease. The general symptoms of anæmia are, general muscular weakness; weakness of the heart, as is shewn by the pulse; feebleness of the whole circulation, manifest in the coldness of the extremities; weakness in the organic functions, shewn by loss of appetite, indigestion, torpor of the bowels, scanty and disordered secretions, defective nutrition, and imperfect sanguification. The physical signs of anæmia are: paleness of the surface, as also of the lips, gums, and tongue. In the course of the larger veins, especially the jugulars in the neck, the thin blood running with great rapidity in the ill-filled vessels, is often thrown into sonorousness, vibrations (venous murmurs) sometimes sensible to the finger placed lightly on the vein. The blood, when drawn, is very thin and watery, it coagulates readily, and forms a very small contracted clot, generally covered with a buffy coat. Andral considers this appearance due to a predominance of the fibrin over the red particles. The albumen is often scantier than usual, chiefly in those cases attended with dropsy. Anæmia is not unfrequently accompanied by symptoms indicating irritation or exaltation of function. Some of these arise indirectly from weakness, as pain, nausea, colic, and diarrhœa, traceable to weakness of digestion. Various properties of the nervous system are sometimes exalted; sensibility is acute; intolerance of light and sound, with flashes in the eyes, noise in the ears, a sense of rushing in the head, and various neuralgic pains. The excito-motory nerves are also sometimes excited, and spasms or convulsive affections may be present, or the organic functions may be affected, and palpitation, spasmodic asthma, vomiting, &c. occur. Thus the functions sometimes excited in the midst of general depression and weakness, are those of the nervous centres. Dr. Williams attempts to account for this by the peculiar distribution of the circulation through the nervous centres. When the blood is reduced in quantity, the blood-vessels, by reason of their tonicity, contract in proportion. But the vessels within the scull and spinal canal cannot contract with the same facility; for not being subjected to atmospheric pressure, they do not shrink as the blood becomes reduced, and therefore they retain more than their due share of the circulating fluid. This disproportionate amount of blood in the nervous centres produces different effects, according to the degree in which the heart's propulsive power reaches it. Under the influence of excitement, the brain and spinal cord receive, through their uncontracted vessels, an unusual share of the force from the heart; hence arises an erethism of some one or other of the functions of these parts; this occasions pain, spasm, sensorial disturbance, or sympathetic irritations of some kind or other. On the other hand, if the heart's action is weak, the blood may stagnate in the vessels of the brain, and produce symptoms of congestion there. Hence head-ache and giddiness, drowsiness, impaired mental faculties, and, in extreme cases, coma or catalepsy. In such cases the blood is accumulated in the veins and sinuses of the brain—even a coagulation of the blood may take place in the sinuses. Dropsical effusion into the cellular texture is a common result of anæmia, when either long-continued, or aggravated by other causes, disturbing the circulation. The exciting causes of anæmia are the various circumstances which abstract blood from the system, or prevent its healthy formation. Irregularity of the uterine function is, however, one of the commonest causes. The *remedial measures* for anæmia are soon told—a nourishing diet—tonics suited to the particular case—exposure to pure air and to light. With respect to tonics, iron holds the very first place; the best form, according to Dr. Williams, is the iodide of iron, in solution with syrup. Where iron disagrees, milder tonics, as calumbo and other bitters, with mineral acids, answer better at first. After excessive losses of blood, sulphate of quinine may be given with the iron.

We now come to the consideration of *Hyperæmia*, which is an extremely frequent element of disease. This state implies undue distention of the containing vessels; with respect to the vital properties of these vessels, and of the heart, scil. tonicity and irritability, hyperæmia has been divided into active or sthenic, and passive or asthenic. Hyperæmia may be either general, or local. General hyperæmia may arise either from too much blood being made, or too little being expended. In either case the blood accumulates and fills the heart and blood-vessels inordinately. The causes inducing plethora are the very reverse of those which cause anæmia. Besides the ordinary causes, the diminution of an habitual excretion or loss of blood, the drying up of an old sore or issue, or the loss of a limb, may occasion this state.

The division of plethora into *sthenic* and *asthenic* is founded on different proportions of the strength and irritability of the moving fibre. Sthenic plethora is that which commonly affects the young, the active, and those of a sanguine temperament. The tendency is to cause general febrile excitement, active hæmorrhages, fluxes and inflammations. In asthenic plethora there is in general want of contractility and tone in the moving fibre. The heart, instead of being excited, is oppressed by the increased quantity of blood. Its functions, in general, are sluggish and imperfectly carried on. This form of plethora affects more especially those weakened by age, excesses, or previous disease, and those in whom the excreting organs act imperfectly. Asthenic plethora tends to produce congestions and passive hæmorrhages, and fluxes or dropsies; and also dilatation of the heart, enlarged liver, varicose veins, &c. Congestion of the brain, with apoplexy or palsy, is sometimes produced.

*Remedies.*—These consist chiefly in blood-letting, and other evacuants, with abstinence. After blood-letting the pulse becomes softer, weaker, and less frequent in the *sthenic* kind; whilst, in the *asthenic*, it often improves in strength and regularity, and sometimes rises to its natural frequency. The secretions must be duly attended to in both kinds of plethora. In the *sthenic* form, sedative and relaxing remedies are also indicated. In *asthenic* plethora, the use of tonics should be combined with blood-letting. The continued use of alterative aperients and diuretics, with taraxacum, nitric acid, iodide of potassium, &c. may prepare the way for the employment of tonics.

We now pass on to *Local Hyperæmia*, with diminished motion, or, in other words, *congestion*, which the author defines to be excess of blood in the vessels of a part, with diminished motion of that blood. Blood-vessels become congested or unduly dilated, when their property, elasticity, or tone is overcome. The chief causes of congestion may be classed under these two heads: 1. *Those of venous obstruction;* and 2, *those of atony of the vessels* (capillaries and veins.) Instances of congestion from venous obstruction are of frequent occurrence, both externally and internally. When the arm is tied for venæsection, congestion is produced. Congestion of brain may be produced by a tumor pressing on the jugular veins. Disease of the valves of the heart, which prevents the blood from passing onwards through it, produces fulness of the veins and of the capillaries in both the pulmonic and systemic circulation. Obstruction to the transit of blood through the liver causes congestion in the abdomen, hemorrhoids, &c. Emphysema of the lungs, in which the efforts of expiration predominate over those of inspiration, occasions congestions not merely by opposing the return of blood through the veins into the chest, but also by removing that suction influence, which naturally promotes the flow of blood in that direction at each inspiration. It is now known that the circulation in the liver is, in health, much dependent on this influence; the diminution of this influence by extensive vesicular emphysema will assist in explaining why hepatic congestion is so commonly combined with this pulmonary lesion.

*Congestion from Atony of the Vessels.*—Sometimes the atony of the vessels is general, being caused by extreme debility from any cause—the blood thus accumulates in some of the vessels, chiefly those that are lowest in the position of the body. In other cases the weakness is local, and is produced by *over-distention.* Over-excitement of the vessels is another cause of congestion. Thus, after a part has been inflamed, the vessels often remain dilated. Congestion occurs in various organs and surfaces, when their proper secretions are arrested, or suddenly diminished. It is not easy to determine whether the congestion is the effect or the cause of the defective secretion in the first instance; very probably the relation is mutual. Atony of the small vessels has now been considered as a chief cause of congestion, not only by making them yield, and become distended by the accumulation of blood, but also by rendering them unfit to transmit the force of the current in its proper direction. Vessels, after losing their tone, become inelastic and tortuous, and, by the very stagnancy of the blood in them, they oppose an encreasing obstacle to its passage through them. The physical principle here referred to, the author illustrates by some experiments. These experiments, according to the author, serve to illustrate a principle that is too little considered in animal and general physics: the *loss or neutralization of force by misdirection.* The blood-vessels, in their healthy condition, are so constituted as to make the most of the hearts' propulsive power and transfer it throughout their whole length; but when dilated, tortuous, flaccid and otherwise altered, they misdirect and exhaust it.

*Symptoms and effects of Congestion.*—Simple congestion generally impairs the vital properties of internal organs. Natural contractility and sensibility are lowered, but pain, spasm, and morbid sympathies are frequently excited. Thus congestion of the liver is sometimes accompanied with pain or tenderness; sometimes it is without either. Congestion of the stomach sometimes causes gastralgia, nausea, vomiting and altered appetite; yet these symptoms are often absent. The same remark is applicable to other organs. The natural secretions from congested parts are at first augmented, as in congestion of the conjunctiva; but generally they are diminished, as bronchial congestion (dry catarrh), and congestion of the liver, kidneys, &c. Congestion often leads to an increased transudation from the whole distended capillaries, causing effusions of the watery and saline parts of the blood, as is seen in the fluids of fluxes and dropsies. The process by which this is the effect of congestion or secretion seems to be chiefly a physical one. Thus the more essential effect of congestion is to impair the natural secretion. The distention of the more congested capillaries sometimes leads to a general exhalation of their more watery contents, which, mingling with the natural secretion, render it watery and sometimes albuminous. Thus congestion of the intestines may produce diarrhœa; congestion of the kidneys, watery and sometimes albuminous urine; congestion of the lungs and pleura, hydrothorax; of the heart, hydropericardium; of the abdomen, ascites, &c. The element of congestion chiefly concerned in producing these effusions is distention of the vessels. These effusions more usually result from congestions occasioned by venous obstruction, especially when these occur suddenly, the vigour of the circulation not being impaired. It may be well to mention that, besides distention of the vessels, the state of the blood considerably influences the result. Where the blood is poor, the watery parts easily pass from congested vessels, and contain but little albumen. But if the blood is rich, abounding in proteine compounds, more pressure is required. Fluxes arising from congestion of high tension exhibit an unusual amount of animal matter of an albuminous or mucous kind. The author states that he has, for several years, referred albuminous urine to congestion of the kidney, for the following reasons: 1. The urine often becomes albuminous during great embarrassment of the circulation in cases of organic disease of the heart, when the kidneys are otherwise healthy. 2. He has, in a few instances, observed temporary albuminuria during the congestive stage of eruptive fevers. 3. In granular degeneration of the kidney, the amount of albumen in the urine is augmented by circumstances causing congestion of the kidney, and is removed by remedies suited to remove this. 4. Bright's disease of the kidney, in its earliest stage, presents the appearance of a highly-congested structure, and is excited by causes calculated to produce congestion, such as frequent irritation of the kidneys by stimulating liquors—congestion from exhausted tone; continued exposure to cold, especially after the kidneys have been thus excited—congestion from intropulsion. 5. The albumen in the urine abounds most in the congestive (first) stage of Bright's disease. We now shall pass in review the various *remedies* for congestion. The most important of these are such as contribute to the removal of the causes. Thus, when the congestion arises from the various forms of venous obstruction, such obstruction is to be removed by suitable means, or by repressing inordinate action of the heart, restoring the secretion of the various organs, &c. In the treatment of congestion arising from atony or weakness of the capillaries, the circumstances which have caused it, must be removed. Pressure, by supporting the weak vessels and promoting their contraction, is sometimes effectual in removing congestion—as also friction, astringents, and stimulants. Under certain circumstances congestion is better relieved by depletion and other evacuants. In general, congestion being in many instances caused by atony of the vessels, it may be counteracted by astringent and stimulant applications, which brace the fibres and invigorate the circulation in a part —general tonics operate in a similar way on the whole system. It is probably in this way that bark and arsenic act—by their power of augmenting the tone of the vessels, they both prevent and remove internal congestions in ague. Iodine and its preparations seem to possess a similar virtue. Mineral acids have a like effect on general weakness.

We next come to the interesting subject of LOCAL HYPERÆMIA—*with motion encreased.* This state is also called *Determination of Blood*—of this we have numerous instances even in health; as blushing: the state of the uterus and breasts, at the periods of gestation and lactation, are the seats of determination of blood, as is evident by the encreased quantity of blood in the part, and by the stronger pulsation of the arteries leading to the part. Determination to the head is a familiar instance: this is characterized by enlargement and throbbing of the temporal arteries. Fits of epilepsy and convulsive hysteria are immediately preceded by throbbing of the carotids, shewing that determination of blood is the proximate cause of the paroxysm. Pressure on the carotids has been known to prevent convulsive fits. The most common cases of determination of blood are those caused by the application of stimuli. In answer to the question, what is the physical cause of determination of blood? the author answers, that it is effected by enlargement of the arteries, which enlargement is the effect of the pressure of the arterial distention from behind acting on a tube, which has lost some of its contractile power. Thus the enlargement of the arteries leading to a part is the physical cause of determination of blood to that part. But to account physiologically for the cause of this enlargement, is not so easy a matter. The terms "active dilatation" (Hunter), and "vital turgescence" (Kaltenbrunner), have been applied to this state. The phrase "active dilatation," however, as applied to arteries, seems to be a contradiction in terms. According to the author's views, the physiological condition seems to be a diminution of tonicity in the artery; so that it becomes passively distended by the *vis a tergo*. Dr. Billing's explanation of this diminished tonicity is, that it is occasioned by abstraction of the nervous influence from the dilated vessels. To this however Dr. Williams objects, for this, among many other reasons, because it assumes that muscular irritability, even in its lowest form, tonicity, is a property derived from the nerves. The objection, however, appears to us to be totally devoid of force—in the first place, we cannot help thinking that between muscular irritability and arterial tonicity there is a difference, somewhat more than in *degree*, we think there is a difference and a well-marked difference, in *kind* also—and again, even admitting that the property called tonicity was not exclusively dependent on nervous influence, no one will attempt to say that it is totally unconnected with it, or independent of it. But, as the author justly observes, the laws of tonicity, and its relation to the nervous influence, require further investigation. The *final* cause of determination of blood is, that it is intended to support the well-being and function of the part, on the principle, "ubi stimulus, ibi fluxus." Determination of blood to internal organs may be occasioned by the application of cold to the surface of the body; for, by constricting the vessels of the surface and extremities, the force as well as the quantity of the circulating fluid are thrown on internal parts. Thus cold weather will cause dyspnœa, pain in the chest, pains in the head, colic, &c.

We shall now pass on to consider some of the results of general and local hyperæmia, scil. hæmorrhage, flux, and dropsy. The blood-vessels, when distended to a great degree, sometimes give way, and blood is effused. Congestion from *venous obstruction* produces hæmorrhage in the cases of *pulmonary apoplexy*, from obstruction on the left side of the heart; bronchial hæmorrhage and *hæmoptysis* from tubercles in the lungs; hæmatemesis and bleeding piles from obstructions of the liver. All cases of general or local hyperæmia do not result in hæmorrhage; some additional element is wanting; this element may be either in the *blood-vessels* or in the *blood*. Sometimes the blood-vessels are in a diseased state from various causes—from various deposits into them—softening from inflammation or mal-nutrition, and from ulceration—and sometimes they are ruptured by mechanical injuries. In other instances the hæmorrhagic disposition may be traced to a diseased state of the blood. Another result of various kinds of hyperæmia is an effusion of the watery part of the blood with more or less animal and saline matter in solution. This result occurring in secretory organs or open surfaces, constitutes *fluxes;* in closed sacs or cellular texture, it constitutes *dropsies*. We shall first consider what fluxes and dropsies have in common. General plethora sometimes ends in flux or dropsy—the same result may happen when the blood-vessels are temporarily distended with an undue proportion of watery contents, as by injecting water into the veins of an animal; or by copious drinking of any liquid, especially where the functions of the kidneys or skin may be suspended from any cause. The most common causes of venous obstruction are visceral diseases, and these commonly produce either dropsy or flux. Thus, cirrhosis of the liver is the most frequent cause of simple ascites. Structural diseases of the heart, especially if they affect the orifices or valves, commonly cause hydrothorax, bronchial flux, and sometimes general dropsy. Pulmonary congestion from causes impeding the respiration, sometimes results in bronchorrhœa or hydrothorax. Dropsies and fluxes may proceed from weakness of the circulation and atony of the vessels. Fluxes and dropsies sometimes occur after previous excessive excitement of the vessels of a part. Flux and dropsy are sometimes found to succeed to one another—ascites may subside on the occurrence of diarrhœa, or may come on when a diarrhœa of long-standing has been suddenly checked. Besides the causes already assigned, fluxes and dropsies may be traced sometimes to a general lax, flabby state of the tonic and contractile fibre, or to a poor, watery state of the blood, or to both.

We have seen that, of all the conditions of the blood tending to watery effusion, a poor or watery state of this fluid is the most obvious—it is for this reason that persons who have lost much blood are so liable to become dropsical; the bulk of the lost blood is replaced by watery serum absorbed from various sources; and thus the blood is in a diluted state. The way in which watery blood tends to produce dropsy and flux is not merely by the greater proneness of thin fluids to transude through the walls of the vessels, but also by the failure and irregular distribution of the force of the circulation, occasioned by the circulation of such blood. The circumstances which induce the thin state of the blood in its relation to dropsy, may be chiefly referred to imperfect excretion by the kidneys, liver, and skin, as the most common cause. In various forms of hyperæmia leading to dropsy and flux, these results generally ensue in proportion as the excreting organs fail, and their removal of these results is to be effected chiefly by means which restore or compensate the defective excretion. Exposure to cold may be followed by dropsy, and this result may appear attributable to checked perspiration; but checked perspiration of itself will not do it; there must be a failure in the action of the kidneys before this result will ensue. The occurrence of dropsy after scarlatina our author accounts for thus: it is observed in all such cases that the urine has been found albuminous, a circumstance which shews that the diseased action of the kidney is the most essential lesion connected with general dropsy—our author thinks that scarlatina impairs the action of the kidney by causing in these glands a highly congested state which injures their secreting power. The form of dropsy which has been called inflammatory, from the circumstance of its being accompanied by a febrile state of the system, is a frequent result of exposure to cold. This inflammatory character of dropsy our author accounts for in a very ingenious way, by referring it to the irritating quality of the excrementitious matter which the failing function of the kidney leaves in the blood. Under such circumstances, urea has been found in the blood and in various effusions, and may be regarded as the *materies morbi* which irritates various parts, and from which the system seeking to relieve itself, excitement and various discharges ensue. This affection resembles acute rheumatism in two points; 1, in the number of parts which may be simultaneously or successively affected, and in the want of any constancy in the seat of the affections. Both these points prove that that the cause is essentially situate in the blood. The nature of the excrementitious matter which accumulates in the blood, also approximates these affections to gout and rheumatism. In the latter affections we know that lithic and lactic acids chiefly constitute this matter; and in these same affections there is very little doubt that urea is either produced in excess, or insufficiently excreted. We should not forget the proximity in composition between lithic acid and urea, and the probable conversion of the former into the latter. Lastly, the connexion between scanty urine and gout and rheumatism is apparent from the fact that rheumatism is frequently complicated with albuminuria (as after scarlatina); and granular degeneration of the kidneys (Bright's disease) is apt to supervene in the most aggravated forms of rheumatism. Besides the retention of excrementitious matter in the blood, there is a loss of albumen from this fluid. This, by thinning the blood, probably facilitates dropsical and profluvial effusions, more especially in the more chronic cases, and in the most anæmic subjects. Thus, then, it may be inferred that acute dropsy arises chiefly from the retention in the blood of excrementitious matter and water, which the kidneys fail to eliminate; and that the more chronic kinds, although often originating in the same way, are rather dependent on a poor or watery state of the blood, especially deficient in albumen. We shall now present as succint a view as possible of the *treatment of dropsy*.

Besides the means required to remove the variety of hyperæmia inducing the dropsy, we must remedy those conditions of the blood which specially favour its occurrence. We have seen that a failure in the secreting powers of the kidneys is the chief cause of these conditions. This failure we have seen is chiefly owing to a highly congested state of the kidneys, which induces albuminuria and its consequences. Thus then inflammatory or acute dropsy after scarlatina or exposure to cold is to be treated by blood-letting (cupping to the loins), hydragogue purgatives, and diaphoretics at first; afterwards by diuretics, which promote the action of the kidneys. Mercury has been found peculiarly efficacious in dropsy connected with diseased liver; and in combination with squill, digitalis, henbane and conium, forms the most useful diuretic in all recent cases of dropsy dependent on congestion without disease of the kidneys. In asthenic dropsy connected with a watery state of the blood, nourishing diet and tonics are indicated. Where dropsy is connected with long continuance of structural disease of the kidneys, liver, or other organs, tonics and invigorating measures must be combined with means to excite the failing excernent organs, or to produce some compensating discharge. Thus, in dropsy from chronic albuminuria, or advanced degrees of granular degeneration of the kidney, the occasional exhibition of hydragogue purgatives and diaphoretics and of diuretics (cantharides, digitalis and colchicum), is useful at the same time that bitters, with iodide of potassium, or mineral acids, are given to support the strength. In the more anæmic cases, iron is of use, unless it should be found to impair the little secreting power left in the kidneys, or to render the urine albuminous. Asthenic dropsy connected with diseased liver is often much relieved by mercurial and diuretic medicines, followed by or conjoined with vegetable tonics. The tendency of dropsy connected with disease of the heart, kidneys, or liver, to recur, and become chronic, renders it needful to vary as much as possible the remedies employed, as well as to support the strength. In such cases we must not exhaust the powers of any secreting organ by too long acting on it, nor should we expend the efficacy of any one remedy by too long continuing its use. By employing sometimes diuretics, sometimes purgatives, sometimes diaphoretics, and by aiding each of these by local depletion or derivants, or by stimulants and tonics, according to the temporary prevalence of vascular fulness and excitement, or the converse, much may be effected to prolong life. It is useful, under such circumstances, to have at command a great variety of medicines, particularly diuretics, and to alternate them or vary them, in order to maintan, or increase their effects. Those found by the author most effectual are—combinations of mercury, squill, digitalis, and conium, (not in acute albuminuria); combinations of decoction of broom, or pyrola umbellata, with nitrate and acetate of potash; the juice or extract of taraxacum, with the same salts or bitartrate of potash, or with nitric acid, (particularly in hepatic disease); infusion or tincture of digitalis, with iodide of potassium, and bitartrate of potash (in dropsy after scarlatina); the same, together with increasing doses of tincture of cantharides (in asthenic cases of albuminuria, after cupping to the loins and hydragogue purgatives); ammonio-tartrate and ammonio-citrate of iron in Seltzer water, (in asthenic dropsy); gin in cream of tartar beverage (imperial); compound spirit of juniper, spirit of nitric æther, with various others (in cases of debility).

The subject of *inflammation* next presents itself; but to attempt an analysis of it here, would be entirely out of the question. We must therefore refer the reader to the book itself, assuring him that he will find his advantage in an attentive perusal of it. We shall now proceed to an analysis of that portion of the work which treats of Diagnosis and of the different modes of death. Diagnosis, says our author, may relate to diseases in their essential nature or pathology, or to those groups of symptoms that are classed as separate diseases by nosological arrangements. In other words, the object of diagnosis is to determine either the intimate nature and seat of a disease, or its name and place in some nosological arrangement. Diagnosis may be *general* or *special*. *General* diagnosis comprehends the distinction between the principles or elements of disease, as, for example, between congestion and inflammation; between nervous irritation and structural disease, &c. This is properly a branch of general pathology. *Special* diagnosis relates to the distinction of diseases according to their chief seat, where they have one, or, according to some other specific difference, where they have no particular seat. The modes of distinguishing diseases will vary much in different cases, according to the class of symptoms which first present themselves. This is illustrated by the following problems: general pathology having pointed out the general nature of a disease, it is required to determine its precise seat.

*Example.*—In a case in which fever, hard pulse, buffed blood, and local pains indicate inflammation, the seat of the inflammation is determined by the chief place of pain or uneasiness, (in the chest or side,) by the function most disturbed, (difficult breathing and cough,) to be in the organs of respiration; by the secretion proceeding from the part, (rusty, viscid expectoration,) and from the physical signs, (impaired breath-sound and stroke-sound in part of the chest with crepitant rhonchus,) to be in the parenchyma of the lungs; that is pneumonia. General pathology here commences the diagnosis, which is completed by reference to symptoms explained by physiology and special pathology.

With respect to *Prognosis*, or the fore-knowledge of the results of disease, may be either *empirical* or *rational*. *Empirical* prognosis is that which is founded on experience or observation only, without regard to the nature of the disease or the reasons which determine the results. Rational prognosis is the estimation of the importance and tendencies of a disease from a knowledge of its causes, its true nature and symptoms, and of the power of treatment in regard to it. The chief circumstances from which a rational prognosis may be formed are:—the cause of the disease—the age—sex—temperament—present diseases and previous habits of the patient—state of patient at the time of the attack—seat and nature of the disease—its extent and progress—character of the symptoms. Bad symptoms are those which arise from an impediment of one or more of the functions more immediately concerned in the sustenance of life, the circulation of the blood, respiration, nutrition, and excretion. In proportion as these functions are more or less interfered with, life is threatened, and there is an approach to its destruction by one or other of those terminations, called *modes of death*. Thus there is death by *syncope*—cessation of the circulation; by *asphyxia* or *apnœa*—interruption of the respiration; and by *inanition*—death *by the pernicious influence of excrementitious matters*, and by poisons. All these agree in affecting the blood, either by altering its composition, or by arresting its circulation.

Death by *cardiac syncope* may occur in two ways—1, by the heart losing its irritability—2, by its being affected with tonic spasm. In both cases death is instantaneous, the patient suddenly turning pale, falling back or dropping down, and expiring with one gasp. The diseases in which death by cardiac syncope sometimes takes place are those of the heart; hæmorrhagic apoplexy; anæmia and adynamic fevers. Death by the *gradual cessation of the heart's action* is called *asthenia*—this is the mode of termination of many diseases, those which destroy life by exhausting the strength. The symptoms of the approach of death in this way, are those indicative of progressive loss of power. By *asphyxia* or *apnœa* is understood that mode of death wherein the respiratory function is the *first* to fail. Death by simple apnœa occurs in diseases of the lungs or air-tubes, in which the entrance of air into the lungs is impeded by any cause. Death by *coma*, or beginning at the brain, is caused by various influences —by obstruction to the circulation through the brain by pressure—by coagula within the vessels—by anæmia—and by various narcotic poisons, as opium, alcohol in large quantities, &c. The symptoms of coma are those of interrupted function of the brain, insensibility and suspension of voluntary motion. In conjunction with these symptoms, referrible to the sensorial and voluntary functions, there are often symptoms of various affections of the excitomotory system of the medulla; at first they are those of excitement, such as convulsion, vomiting, hiccup, contracted pupil, &c. The author notices a mode of death to which he gives the name of *necræmia*, or *death beginning with the blood*. This mode is presented in those fatal cases in which the first and most remarkable change is exhibited in the blood. In typhoid, malignant and pestilential fevers, the blood, at an early period, exhibits changes which show that disorder begins with it. The petechiæ and vibices on the external surface, hæmorrhages in internal parts, its fluidity and unusually dark aspect, its proneness to pass into decomposition, &c. all point out the blood as the first seat of disorder.

We find, from the length to which our analysis of this work has run, that we must now conclude. The extent of this analysis sufficiently indicates the high estimation in which we hold the work. We consider, in fact, that in producing it Dr. Williams has still further enhanced his already high character with the profession and the public. The plan of the work, the simplicity of style in which it is written, the happy illustrations in proof of the various positions laid down in it, derived from various sources, as also from his own extensive practice, all contribute to render it one of the most valuable boons conferred for many years on the student of medicine. We should like to have seen in it a general pathology of the different tissues and structures of the body, somewhat on the plan of Bichat's General Anatomy—or rather something like Pinel's arrangement of the diseases affecting the various membranes and tissues, the work, by the way, which first suggested to Bichat the composition of his celebrated *Anatomie General.* We merely throw out this hint for Dr. Williams' future consideration in preparing a new edition, which we feel no donbt will soon be called for.

